# The Pneumococcus

**DOI:** 10.3201/eid1102.041010

**Published:** 2005-02

**Authors:** Cynthia G. Whitney

**Affiliations:** * Centers for Disease Control and Prevention, Atlanta, Georgia, USA

**Keywords:** Streptococcus pneumoniae, pneumococcus, book review

*Streptococcus pneumoniae*, known as the pneumococcus, remains an important pathogen in spite of advances in medical care. Globally, as many as 1 million children die of pneumococcal infections each year, nearly all in developing countries. Pneumococcal disease is also common in children in industrialized countries, although in those settings nearly all such deaths occur in older adults or adults with chronic medical conditions. Given its place near the top of the list of killer bacteria, pneumococcus is a focus of numerous researchers around the world. A new book, The Pneumococcus, edited by Elaine Tuomanen et al., is the latest effort to summarize the state of research on the organism.

The book begins by providing a well-thought-out answer to a basic question—what is a pneumococcus?—and moves on to chapters on topics ranging from attachment and invasion of the respiratory tract to vaccine-induced immunity. The editors are leaders primarily in the areas of molecular biology and pathogenesis, and the focus of much of the book is on these topics, although issues such as treatment, carriage, disease in persons with immunodeficiencies, antimicrobial resistance, and epidemiology are also well covered. The relatively recent deciphering of several pneumococcal genomes has led to a new outburst of research activity, aspects of which are summarized in several of the chapters.

All of the authors are recognized experts in their respective areas. The foreword by Robert Austrian, a pioneer in pneumococcal microbiology, disease description, and vaccine work, provides an interesting summary of the history of major discoveries in the field. While covering many areas of pneumococcal research, the book is not exhaustive; for example, issues specific to pneumococcal disease in developing countries are mentioned only in passing.

The book may be most suitable as a tool for new researchers in the pneumococcal field, but it may also be useful for medical students, graduate students, and infectious disease specialists. The level of detail varies among the chapters, but it is adequate to provide an introduction to each of the topics covered, and all chapters are thoroughly referenced. Overall, the editors and writers have done a remarkable job of consolidating the latest information. The Pneumococcus is an authoritative reference in a rapidly changing field.

